# What causes increasing and unnecessary use of radiological investigations? a survey of radiologists' perceptions

**DOI:** 10.1186/1472-6963-9-155

**Published:** 2009-09-01

**Authors:** Kristin B Lysdahl, Bjørn M Hofmann

**Affiliations:** 1 Faculty of Health Sciences, Oslo University College, Oslo, Norway; 2 Section for Medical Ethics, Faculty of Medicine, University of Oslo, Oslo, Norway; 3 Faculty of Health Care and Nursing, Gjøvik University College, Gjøvik, Norway

## Abstract

**Background:**

Growth in use and overuse of diagnostic imaging significantly impacts the quality and costs of health care services. What are the modifiable factors for increasing and unnecessary use of radiological services? Various factors have been indentified, but little is known about their relative impact. Radiologists hold key positions for providing such knowledge. Therefore the purpose of this study was to obtain radiologists' perspective on the causes of increasing and unnecessary use of radiological investigations.

**Methods:**

In a mailed questionnaire radiologist members of the Norwegian Medical Association were asked to rate potential causes of increased investigation volume (fifteen items) and unnecessary investigations (six items), using five-point-scales. Responses were analysed by using summary statistics and Factor Analysis. Associations between variables were determined using Students' t-test, Spearman rank correlation and Chi-Square tests.

**Results:**

The response rate was 70% (374/537). The highest rated causes of increasing use of radiological investigations were: a) new radiological technology, b) peoples' demands, c) clinicians' intolerance for uncertainty, d) expanded clinical indications, and e) availability. 'Over-investigation' and 'insufficient referral information' were reported the most frequent causes of unnecessary investigations. Correlations between causes of increasing and unnecessary radiology use were identified.

**Conclusion:**

In order to manage the growth in radiological imaging and curtail inappropriate investigations, the study findings point to measures that influence the supply and demand of services, specifically to support the decision-making process of physicians.

## Background

Utilization of high-technology and high-cost diagnostic imaging has increased substantially over the past decades [1-7]. This growth can be attributed to various factors such as aging populations, advances in imaging technology, that radiology is indicated in more clinical conditions [6,8], availability of the technology [9] and increasing number of radiologists [10]. Referring physicians have a central role in how radiological services are used, and studies have singled out several factors affecting their test-ordering behaviour, including patients' expectations [11-14], professional uncertainty [12,14], stress from uncertainty and time constraints [15], defensive medicine [16,17], payment system [18], and physicians' self-referral [19,20]. The significance of these factors may vary by institutional structures and across countries.

Some of these influences may lead to utilization growth as well as over-utilization [21]. Over-utilization implies wasteful investigations, which according to European Referral guidelines for imaging [22] has the following main causes: repeating investigations, investigations when the results are unlikely to affect patient management, investigating too often, doing the wrong investigation, insufficient clinical information and unclear referral questions, and over-investigation (for reassurance of clinicians and patients). Over-utilization is the main concern among the problems of inappropriate utilization [23].

The expansion of radiological services has a significant impact on health care costs [23,24], the quality of health care services and health care risks [10]. The risk of radiation exposure is drawing increasing attention [25,26]. Because some reasons for expanded use may not benefit patient care, it is crucial to identify the main factors contributing to imaging growth.

Although many influencing factors are known, few studies have explored and quantified their relative impact on the use of diagnostic imaging, and hardly any have studied the subject from the position of those who provide the services. Radiologists are in a key position to illuminate this topic as their perception is refined through experiences with a multitude of referrals, interaction with clinicians and patients, and knowledge of indications for imaging. Their perceptions of the mechanisms behind increased and unnecessary use of radiological investigations can provide important input for managing the growth of imaging and limit over-utilization. The purpose of this study was to obtain radiologists' perception of causes of increasing and unnecessary use of radiological investigations.

## Methods

### Setting and study population

This was a national survey conducted in Norway, where radiology services are provided by public in-house state-run hospital departments and by private radiology institutes. Both receive public refunds for ambulatory services, and Norway has universal health coverage. (See Additional file 1 for further information). Data on radiologist members were obtained from the Norwegian Medical Association (NMA), where almost all (96.8%) physicians are members [27]. Their lists contain all currently practising members, both approved specialists and registered trainees in radiology (registrars). In February 2007 this totalled 564 physicians (not including those with addresses abroad) who were invited to participate in the survey. The introductory letter to the survey questionnaire informed them of the purpose of the study and the confidential handling of their responses. The study had approval from the Norwegian Social Science Data Services (NSD).

### Survey

The questionnaire was constructed after an extensive literature review, as no suitable tool was found. Survey validity was tested by group and individual interviews with radiologists from four different practice settings. This led to changes in content and format and reduced the length of the questionnaire. Further minor adjustments were made based on responses from an anonymous pilot survey sent to 20 physicians randomly selected from the list of 564.

In mid April 2007 the remaining 544 radiologists/registrars in radiology were sent the final questionnaire with an introductory letter and a return envelope with postage paid. Four weeks later, a reminder was sent with a new copy of the questionnaire and a new return envelope. No respondent identifier was applied, hence the reminder was sent to all individuals.

The questionnaire consisted of four parts; this study is based on parts 1 and 4 (see Additional file 2: The original questionnaire). Part 1 contained two questions regarding use of radiological investigations. Question 1 read: "The volume of radiological investigations is increasing in Norway. To what extent do you think this may be caused by the following factors?" Fifteen possible causes (items) were listed. Respondents could also provide additional causes in free-text. Question 2 listed the six main causes of unnecessary use of radiological investigations, as stated in Referral guidelines for imaging [22], and asked "To what extent do you think this occurs at your workplace?" In both questions a five-point response scale (to a very small extent, to a small extent, to some extent, to a large extent, to a very large extent) was used. To ease interpretation and presentation of the distribution of responses, the two responses at each end of the scale were combined in the analyses.

Part 4 of the questionnaire concerned demographic, professional and practice setting characteristics of the respondents. It also contained questions about local access to radiological services (capacity at own workplace, travel time to closest other provider of radiology services).

### Data analysis

Descriptive analyses were applied to the entire sample of respondents to examine demographic, professional and practice setting characteristics variables (listed in Additional file 3) by frequencies and proportions. Two variables were dichotomized, partly due to the small number of responses in subcategories: *type of institution employed *into the categories of (public) hospitals and (private) radiological institutes, and *capacity of radiology supply in own practice *into insufficient versus sufficient (which includes free capacity). *Years in radiology practice *was categorized into decades.

Frequencies and proportions were also computed from responses to each item in question 1 and 2. To uncover underlying structure in the set of 15 suggested causes of increased investigation volume (question 1) and identify latent themes among the responses, these 15 items were included in an exploratory factor analysis (extraction method: principal components) with varimax rotation (Direct Oblimin rotation did not show strong correlations between the factors). The number of factors to be extracted was based on the Kaiser rule of eigenvalues of > 1.0. Individual items were included in a factor if the absolute value of their factor loading was ≥ 0.4. Sampling adequacy was assessed by Kaise-Meyer-Olkin (KMO) statistics. Finally, internal reliability of the new factors (i.e. latent variables) was measured using Cronbach alpha. Respondents' factor scores were computed as the sum of weighted item scores (raw score on items included in the latent variable multiplied by the item's factor loading).

Associations between factor scores and demographic, professional or practice setting characteristics were analyzed by T-tests (in dichotomy variables) or Spearman rank correlation. Associations between such characteristics and perceived occurrences of unnecessary use of radiological investigations were analyzed by Chi-Square tests (test for trend, using the 3-point scale) and Spearman rank correlation. Associations were calculated with controls for place of employment of respondents (by analyzing hospital radiologists and institute radiologists separately) as respondents employed in hospitals and radiological institutes differed in other background variables. Spearman rank correlation was also used in analyses of correlations between latent variables involved in increased investigation volume and the six causes of unnecessary investigations. *P *values of less than 0.05 (two-tailed) were considered statistically significant. Data analyses and statistical tests were performed using SPSS for Windows (version 14.0) software.

## Results

The overall response rate was 70% (375 of 537 physicians), 276 responded to the first and 99 to the second mailing. The denominator in the calculation was reduced from 544 to 537 because three physicians informed us that they had not responded due to recent retirement, and four questionnaires were returned due to unknown address.

### Respondent demographics and practice information

Eighty-three percent of the respondents were approved specialists in radiology and 57% were men. (The corresponding numbers in the total invited population of 564 radiologists were 83% approved specialists and 59% males.) The mean years in radiology practice was 15.9 years (SD, 10.41 years, ranging from below 1 year to 40 years). The majority of respondents (87%) worked full time in public hospitals (66% in large and 16% in smaller hospitals). The proportion of radiological institute employed radiologists among invited and responders were 9% and 10%, respectively.

One hundred and seventy-two (46%) considered the capacity of radiology supply in their own practice as insufficient, 40% as sufficient and 5% reported free capacity. Travel time to closest other (i.e. neighbour) provider of radiology services was reported to be less than half an hour by 56% of respondents, between half an hour and one hour by 29%, and more than one hour by 9% of respondents.

### Perceived causes of increased investigation volume

Table 1 shows the responses given to the fifteen suggested causes of increased volume of radiological investigations. The five highest scored causes were: increased possibilities due to new radiological technology; peoples' increased demands for certain knowledge about own health; referring physicians' lower tolerance for uncertainty; expanded clinical indications for radiology; and increased availability of radiological equipment and personnel. These causes all received high scores (to a large or very large extent) from 50% or more of the respondents, and low scores (to a small or very small extent) from 10% or fewer of the respondents. Free-text was used by 43 of the respondents mainly to elaborate on given causes rather than to add new ones.

**Table 1:** Radiologists' ratings of the extent to which suggested causes increase the volume of radiological investigations

	Number (%) of responses		
Suggested cause	To a small or very small extent	To some extent	To a large or very large extent
Increased possibilities due to new radiological technology	2 (0.5)	62 (16.6)	310 (82.9)
Peoples' increased demands for certain knowledge about own health	8 (2.1)	95 (25.4)	271 (72.5)
Referring physicians have less tolerance for uncertainty	16 (4.3)	113 (30.4)	243 (65.3)
Expanded clinical indications for radiology	29 (7.8)	128 (34.4)	215 (57.8)
Increased availability of radiological equipment and personnel	37 (9.9)	142 (37.9)	196 (52.3)
Referring physicians have less competence to perform clinical examinations	54 (14.4)	183 (48.9)	137 (36.6)
Increased risk of litigation against health care providers	72 (19.3)	171 (45.7)	131 (35.0)
Increased demand on health care professionals' effectiveness	97 (26.1)	149 (40.1)	126 (33.9)
Strengthening of patient rights	76 (20.5)	192 (51.8)	103 (27.8)
Referring physicians have less knowledge about accurate use of radiology	90 (24.1)	190 (50.9)	93 (24.9)
Increased demands for documentation from the National Insurance Service or insurance companies	129 (34.7)	167 (44.9)	76 (20.4)
Health service providers' increased competition for patients	174 (46.6)	125 (33.5)	74 (19.8)
People's fascination for technological innovations	189 (50.5)	139 (37.2)	46 (12.3)
Increased focus on economic issues in health care services	213 (57.3)	100 (26.9)	59 (15.9)
Increased morbidity in the population	234 (63.1)	114 (30.7)	23 (6.2)

Factor analysis of responses to suggested causes of increased investigation volume identified five latent variables that accounted for 60% of the total variance and embraced causes concerning 1) *referring physicians' uncertainty*, 2) *efficiency and economy*, 3) *patients autonomy and legal claims*, 4) *medical possibilities*, and 5) supply and demand of services - *health market *(Table 2). Reliability analyses showed sufficient internal consistency, according to the convention in exploratory research; Cronbach's alpha above 0.6 [28]. The latent variable *health market *showed low internal consistency and included the item increased morbidity in the population, which was weakly and negatively correlated to the variable. When this item was excluded from the variable, Cronbach's alpha increased from 0.34 to 0.56, indicating that the variable became more likely to measure one single underlying theme. Hence, the item increased morbidity in the population was left out when calculating respondents' factor scores.

**Table 2:** Factor structure and loadings after varimax-rotation^1 ^of causes of increased volume of radiological investigations

Suggested Cause	Referring physicians' uncertainty	Economy/efficiency	Patient autonomy/legal claims	Medical possibilities	Health market
Referring physicians have less competence to perform clinical examinations	0.86				
Referring physicians have less knowledge about accurate use of radiology	0.77				
Referring physicians have less tolerance for uncertainty	0.66				
Increased focus on economic issues in health care services		0.76			
Increased demand on health care professionals' effectiveness		0.76			
Increased risk of litigation against health care providers			0.76		
Strengthening of patient rights			0.70		
Increased demands for documentation from the National Insurance Service or insurance companies		0.47	0.60		
People's increased demands for certain knowledge about own health			0.58		
Increased possibilities due to new radiological technology				0.77	
Expanded clinical indications for radiology				0.77	
Health service providers' increased competition for patients					0.69
Increased availability of radiological equipment and personnel					0.62
People's fascination for technological innovations					0.57
Increased morbidity in the population					-0.56
					
Cronbach's alpha	0.69	0.62	0.61	0.62	0.34
Percentage of variance	18.6%	13.4%	11.1%	9.6%	7.6%

^1^Extraction method: Principal Component Analysis. Only factor loadings greater than 0.4 are displayed.

Factor scores (sum of weighted item scores) for these five new latent variables were weakly associated with a few of the recorded demographic, professional and practice setting characteristics. *Health market *(with factor scores ranging from 3.1 to 9.4) was emphasized more by hospital than radiological institute employees and more by full-time than part-time employees with differences in mean factor scores of 0.79 (95% CI: 0.37, 1.21, *P *< 0.001) and 0.69 (95% CI: 0.19, 1.20, *P *= 0.008), respectively. *Health market *was also emphasized more by those considering the capacity of radiology supply in their own practice as sufficient compared to those considering it as insufficient (mean difference 30.7, 95% CI: 10.0, 51.5, *P *= 0.004). Female radiologists had a higher score on *patient autonomy/legal claims *than male radiologists (mean difference 0.39 in factor scores (that ranged from 4.4 to 13.2), 95% CI: 0.08, 0.70, *P *= 0.012). Finally, approved specialists had a higher score on *medical possibilities *than registrars (mean difference of 0.38 in factor scores (that ranged from 3.1 to 7.7), 95% CI: 0.11, 0.65, *P *= 0.005).

### Perceived causes of unnecessary investigations

Table 3 shows respondents' opinion on causes for unnecessary investigations at their own workplace. The two most frequent causes reported were over-investigation (because some clinicians tend to rely on investigations more than others and some patients take comfort in being investigated), and insufficient clinical information and unclear questions in the referral. As much as 50% and 42% of respondents reported these two reasons, respectively, to occur to a large or very large extent. The least common cause was doing the wrong investigation; 55% reported this to occur to a small or very small extent.

**Table 3:** Radiologists' ratings of the extent to which causes of unnecessary investigations occur at own workplace

	Number (%) of responses^1^		
	
Cause	To a small or very small extent	To some extent	To a large or very large extent
Over-investigation, because some clinicians tend to rely on investigations more than others and some patients take comfort in being investigated	44 (12.3)	136 (38.1)	177 (49.6)
Insufficient clinical information and unclear questions in the referral	57 (16.0)	149 (41.7)	151 (42.3)
Investigation when the results are unlikely to affect patient management, because the anticipated 'positive' finding is usually irrelevant or because a positive finding is so unlikely	97 (27.7)	156 (44.6)	97 (27.7)
Investigating too often, i.e. before the disease could have progressed or resolved or before the results could influence treatment	99 (27.7)	161 (45.1)	97 (27.2)
Repeating investigations which have already been done	138 (38.9)	184 (51.8)	33 (9.3)
Doing the wrong investigation	195 (54.6)	150 (42.0)	12 (3.4)

^1^Only respondents presently working in radiology were asked this question, reducing the maximum number of respondents to 361.

**Figure 1:**
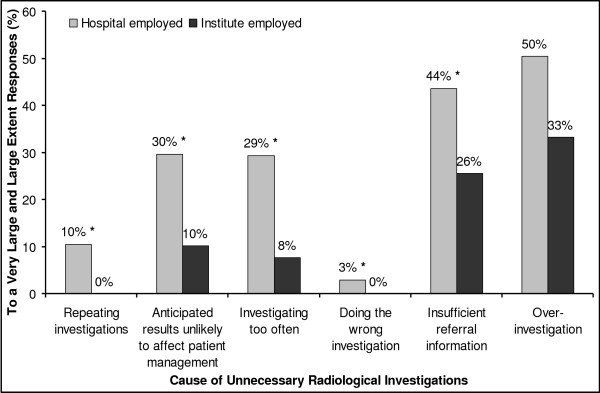
**Hospital and institute radiologists' rating of causes of unnecessary investigations**. Bar graph illustrates radiologist' employed in hospitals (gray bars) and in institutes (black bars) ratings of the extent to which suggested causes of unnecessary radiological investigations occur. Only the responses to a large extent and to a very large extent are displayed (combined). Difference was statistically significant for four of the causes (p < 0.05, Chi-Square test [asterisks]).

Compared to hospital radiologist, radiologist in radiological institutes consistently reported lower occurrence of causes of unnecessary investigations; the difference was statistically significant for five of the six causes (Figure 1). Moreover, single causes of unnecessary investigations differed between subgroups of respondents (after controlling for institution employed). Those who reported insufficient capacity of radiology supply in their own practice reported more repeating investigations (P = 0.003). Male respondents considered investigation when the anticipated result is unlikely to affect patient management to occur more often than did female respondents (P = 0.020). Registrars reported both over-investigation and insufficient referral information to occur more often than did approved specialists (*P *= 0.040 and 0.007, respectively). Finally, reporting insufficient referral information was moderately, negatively correlated to respondents' years in radiology practice (*r *= -0.198, *P *< 0.001).

### Causes of increased volume related to causes of unnecessary investigations

Latent variables embracing causes for increased investigation volume were correlated to causes of unnecessary investigations (Table 4). Respondents who considered unnecessary investigations to occur to a larger extent tended to give emphasis to *referring physicians' uncertainty *and *health market *as reasons for increasing investigation volume. The correlations were generally weak, strongest between *referring physicians' uncertainty *and over-investigation (to reassure referring clinician and patients), but they were statically significant for all six listed causes of unnecessary investigations. Emphasizing *medical possibility *tended to be associated with considering occurrence of unnecessary investigation to be less frequent.

**Table 4:** Correlation between suggested causes of unnecessary investigations and factors involved in increased volume of investigations^1^

	Factor involved in increased volume of investigations				
	
Cause of unnecessary investigations	Referring physicians' uncertainty	Economy/efficiency	Patient autonomy/legal claims	Medical possibility	Health market
Repeating investigations	.220**	-.038	.031	-.066	.239**
Anticipated result unlikely to affect patient management	.321**	.012	.025	-.114*	.179**
Investigating too often	.347**	.029	.016	-.124*	.161**
Doing the wrong investigation	.344**	.018	.039	-.051	.196**
Insufficient referral information	.346**	.092	.035	-.120*	.113*
Over-investigation	.434**	.007	.067	-.090	.196**

^1^Numbers are Spearman Rank Correlation Coefficients.

** P < 0.01, * P < 0.05

## Discussion

Previous studies have focused on selected factors' impact on use of radiology [9,11,19,29,30], or were restricted to clinicians' point of view [12,14]. This study gives new information by suggesting how the diversity of causal factors may be ranked and interrelated, and by presenting the radiologists' perspective. The high response rate and the careful completing of the questionnaire (maximum 3% missing values in single items) indicate that radiologists regard the study topic interesting and important. This impression is strengthened by some feedback (comments in the questionnaires' margin) from the respondents. Focus on their opinions may further increase their awareness of the issue and urge them to become more involved in decisions on the use of their services, which may be warranted [23].

Our findings suggest that the most important causes for increased volume of radiological investigations are: expanded medical possibilities (due to new technology and more/wider clinical indications for radiology), peoples' and referring clinicians' increased demand for assurance (regarding knowledge about own/patients' health condition), and availability of services. This is quite consistent with previous studies of selected causal factors. Utilization of radiological investigations is documented to increase with availability of services, i.e. supply of new technology [9] and distance from clinic to radiology facility site [31]. Improvements in imaging technology are reported to account for much of the increase in investigation volume [24]. Radiologists have more intimate knowledge of supply related factors than demand factors. Their perception of the latter is supported by general practitioners who report that "patients have become better informed about their rights as patients, and that they appear increasingly demanding" and that this affect their referral behaviour [32]. Compliance with patients' requests can be motivated by doctor-patient relationship considerations and by clinical uncertainty [33]. Uncertainty of diagnosis/management and patient pressure are reported the two most commonly agreed factors affecting British GPs' referral behaviour [14]. Self-referrals which is reported to be an important causal factor [21] was not included as an item in the study because virtually all diagnostic imaging in Norway are referred from non-radiologist to radiologists, and performed in radiology departments or radiological institutes [4].

Unnecessary investigations were regarded to occur mainly due to over-investigation and insufficient referral information. Causes that radiologists may better control themselves, such as wrong or repeat investigations, were not emphasized. Studies of referral quality confirm the occurrence of insufficient referral information [34-36], though its consequences are, to our knowledge, not quantified. The radiologists' emphasis on over-investigation corresponds well with studies of the general practitioners' reasons for requesting x-rays, where patient reassurance [37] and own uncertainty [38] are among the important influencing factors. Uncertainty can be related to "ordering criteria, anxiety, skills, or possible legal actions" [38]. Excessive testing can result from physicians being uncomfortable with uncertainty [21] and is a common "assurance behaviour" by practitioners of defensive medicine.

The listed causes for unnecessary investigations were reported to occur less frequently by institute radiologists compared to hospital radiologists. This does not mean that unnecessary investigations actually occur less frequent in the institutes, which is not supported by other research[39,40]. Respondents were not asked about actual occurrence, and other factors than the six listed may be relevant. A plausible explanation may be that a larger proportion of the institutes' patients are in the early stages of disease, which may challenge a strict differentiation between appropriate and the inappropriate requests.

The interrelation between responses to the questions of increasing and unnecessary use of radiology bears some implications. First, *medical possibility *tends to be associated with lower rating of unnecessary investigations. This poses a challenge for management of imaging services, as radiologists may perceive the expansion as inevitable and a good thing. Second, *referring physicians' uncertainty *was associated with higher ratings of unnecessary investigations. This supports the hypothesis that radiologists ascribe the main responsibility for unnecessary investigations to the referring clinician. Consequently, the appropriate remedial actions to reduce over-utilization should be to support clinicians in the decision-making process. Clinicians' insufficient knowledge of appropriate use [41] calls for remedial actions from the radiology community [20].

A limitation of this study is that registrars are underrepresented: 17% in the study sample, whereas nearly 30% in the population [42]. This is because registrars do not inform the NMA about their affiliation (A. Taraldset, NMA, personal information). Accordingly, institute radiologists are somewhat over-represented (3 percentage points), as registrars only work in hospitals. However, more registrars and fewer institute radiologists in the study would most likely have strengthened the findings of over-utilization and insufficient referral information as main causes of unnecessary investigations. Generally, the findings should be treated with caution, as comparable studies are lacking.

It is important to note that our findings are only valid in the Norwegian context. The relative impact of factors influencing use of imaging may vary according to differences in health policies, organization of services, and cultural differences. Nevertheless, similarities in utilisation pattern in many developed countries calls for measures to improve the use of radiological investigations, and certain causes of increased and inappropriate use may be similar in other countries. Hence, the results from our study may be of international interest, to health care administrators, authorities and health policy makers, as well as clinicians and radiologists. Furthermore, our method may be of relevance for other studies.

## Conclusion

According to radiologists' perceptions the most important causes for increased investigation volume are expanded medical possibilities, and supply and demand of services. The latter is also regarded a major cause of unnecessary radiological investigations. This indicates that measures to influence supply and demand of services are important in order to manage growth in investigations volume and reduce unnecessary investigations. Specifically, support to the decision-making process of physicians seems to be important. Further research on factors influencing use of radiology services from the providers' perspective is needed, to confirm, complement or challenge our findings.

## Competing interests

The authors declare that they have no competing interests.

## Authors' contributions

KBL designed the study, collected and analysed the data, and drafted the manuscript. BMH contributed in the design of the study, interpretation of results and in drafting the manuscript. Both authors revised and approved the final manuscript.

## Pre-publication history

The pre-publication history for this paper can be accessed here:



## Supplementary Material

Additional file 1 **Radiology services in Norway**. A brief description of how radiology services are organized in Norway. Click here for file

Additional file 2 **The questionnaire**. The questionnaire in English translation. Click here for file

Additional file 3 **Respondents' demographic characteristics and practice information, and some corresponding number in the population**. The additional file is a table that displays the respondents' replies on demographic and practice setting variables. In addition, the available corresponding numbers in the population (564 radiologists) is provided. Click here for file
