# Supplementary material for: What causes increasing and unnecessary use of radiological investigations? a survey of radiologists' perceptions
Source: BMC Health Serv Res. 2009 Sep 1;9:155. doi: 10.1186/1472-6963-9-155 (PMC2749824; doi:10.1186/1472-6963-9-155)
Supplement: Additional file 1 — Radiology services in Norway. A brief description of how radiology services are organized in Norway. [file 1472-6963-9-155-S1.pdf]

Brief outline of how radiology services are organized in Norway

Radiology services in Norway are provided by public in-house state-run hospital departments and by (private) radiology institutes run by radiologists. The latter provide 28% (figures from 2002) of all services [1], and are restricted to outpatient services. These services have equal terms regarding clinicians' access to them and regarding public refunds for ambulant services. Norway has universal health coverage, and private health insurance is rather unusual. Co-payment by outpatients is equal and small both in the public and in the private setting. In-hospital public services are free of charge.

1. Lysdahl KB, Borretzen I: Geographical variation in radiological services: a nationwide survey. BMC Health Serv Res 2007, 7:21
