# Supplementary material for: What causes increasing and unnecessary use of radiological investigations? a survey of radiologists' perceptions
Source: BMC Health Serv Res. 2009 Sep 1;9:155. doi: 10.1186/1472-6963-9-155 (PMC2749824; doi:10.1186/1472-6963-9-155)
Supplement: Additional file 2 — The questionnaire. The questionnaire in English translation. [file 1472-6963-9-155-S2.pdf]

A Use of radiological examinations

1 The volume of radiological investigations is increasing in Norway. To what extent do you think this may be caused by the following factors?

		To a very small extent	To a small extent	To some extent	To a large extent	To a very large extent
a	Increased morbidity in the population	☐	☐	☐	☐	☐
b	Strengthening of patient rights	☐	☐	☐	☐	☐
c	Peoples' increased demands for certain knowledge about own health	☐	☐	☐	☐	☐
d	Referring physicians have less tolerance for uncertainty	☐	☐	☐	☐	☐
e	Referring physicians have less competence to perform clinical examinations	☐	☐	☐	☐	☐
f	Increased possibilities due to new radiological technology	☐	☐	☐	☐	☐
g	Expanded clinical indications for radiology	☐	☐	☐	☐	☐
h	Referring physician have less knowledge about accurate use of radiology	☐	☐	☐	☐	☐
i	People's fascination for technological innovations	☐	☐	☐	☐	☐
j	Increased availability of radiological equipment and personnel	☐	☐	☐	☐	☐
k	Increased demand on health care professionals' effectiveness	☐	☐	☐	☐	☐
l	Increased focus on economic issues in health care services	☐	☐	☐	☐	☐
m	Health service providers' increased competition for patients	☐	☐	☐	☐	☐
n	Increased demands for documentation from the National Service or insurance companies	☐	☐	☐	☐	☐
o	Increased risk of litigation against health care providers	☐	☐	☐	☐	☐
p	Other, specify: ______________________	☐	☐	☐	☐	☐

If you do not practice as radiologist at present, please jump to question 9

2 The list below shows the main causes for unnecessary use of radiological investigations (Source: European referral guidelines for imaging, in Norwegian translation).
To what extent do you think this occurs at your workplace?

		To a very small extent	To a small extent	To some extent	To a large extent	To a very large extent
a	Repeating investigations which have already been done	☐	☐	☐	☐	☐
b	Investigation when the results are unlikely to affect patient management, because the anticipated 'positive' finding is usually irrelevant or because a positive finding is so unlikely	☐	☐	☐	☐	☐
c	Investigating too often, i.e. before the disease could have progressed or resolved or before the results can influence treatment	☐	☐	☐	☐	☐
d	Doing the wrong investigation	☐	☐	☐	☐	☐
e	Insufficient clinical information and unclear questions in the referral	☐	☐	☐	☐	☐
f	Over-investigation, because some clinicians tend to rely on investigations more than others and some patients take comfort in being investigated	☐	☐	☐	☐	☐

B Participation in the decision-making process

3 How often are you asked about or requested to advice on choice of examination?

		Daily	Weekly	Monthly	Rarer than monthly
a	From the referring physician	☐	☐	☐	☐
b	From the patient	☐	☐	☐	☐

		Yes, often	Yes, sometimes	No, rarely	No, never
**4**	**Do you refer to the Referral guideline for imaging when advising on choices of examination?**	☐	☐	☐	☐

5 Assume that referrals with ambiguous indications or inappropriate choice of technique/modality occur. How often do you carry out the following actions?

		Daily	Weekly	Monthly	Rarer than monthly
a	I check test results and/or information in the medical record	☐	☐	☐	☐
b	I contact the referring physician to clarify the clinical problem	☐	☐	☐	☐
c	I ask the patient for more information	☐	☐	☐	☐
d	I perform a clinical examination of the patient	☐	☐	☐	☐
e	I change technique/modality without contacting the referring physician	☐	☐	☐	☐
f	I return the referral and give a reason for this	☐	☐	☐	☐
g	Other, specify: ________________________________	☐	☐	☐	☐

____

6 Assume that you doubt the usefulness of a requested examination. To what extent would the following factors contribute to you preventing it from being carried out as requested?

		To a very small extent	To a small extent	To some extent	To a large extent	To a very large extent
a	The patient is young (child, youth)	☐	☐	☐	☐	☐
b	The patient/next-of-kin does not want the examination	☐	☐	☐	☐	☐
c	The examination is uncomfortable or a burden to the patient	☐	☐	☐	☐	☐
d	High risk of serious complications or side effects	☐	☐	☐	☐	☐
e	The examination is resource-demanding (time or money)	☐	☐	☐	☐	☐
f	The referring physician is receptive to guidance	☐	☐	☐	☐	☐
g	The examination requires a high radiation dose	☐	☐	☐	☐	☐
h	High risk of false positive/negative findings	☐	☐	☐	☐	☐
i	The radiographer questions the referral	☐	☐	☐	☐	☐
j	Makes one’s work as a radiologist feel meaningful	☐	☐	☐	☐	☐
k	Other, specify: ________________________________	☐	☐	☐	☐	☐

____

7 **Assume that you doubt the usefulness of a requested examination. To what extent would the following factors contribute to you performing the examination as requested after all?**

		To a very small extent	To a small extent	To some extent	To a large extent	To a very large extent
a	The patient/next-of-kin want the examination	☐	☐	☐	☐	☐
b	The patient has already arrived	☐	☐	☐	☐	☐
c	Great respect for the referring physician's professional judgement	☐	☐	☐	☐	☐
d	The referring physician is difficult to contact	☐	☐	☐	☐	☐
e	Ambiguous guidelines for use of the examination	☐	☐	☐	☐	☐
f	High reimbursement rate for the examination	☐	☐	☐	☐	☐
g	Fear of legal consequences	☐	☐	☐	☐	☐
h	Time pressure	☐	☐	☐	☐	☐
i	Demand for efficiency at work	☐	☐	☐	☐	☐
j	Other, specify: ____________________	☐	☐	☐	☐	☐

____

8 **Do any procedural or organisational conditions at your workplace prevent you from influencing decisions regarding what examinations that is to be performed?**

____

____

C Role perception

9 **Some statements concerning radiology and radiologists in general follow below. To what extent do you agree or disagree with these statements?**

		Com-pletely agree	Partly agree	Neutral	Partly disagree	Com-pletely disagree
a	Radiology is a suitable expedient for comforting anxious patients	☐	☐	☐	☐	☐
b	Patients expect the radiologist to become involved in their total clinical situation	☐	☐	☐	☐	☐
c	Radiation doses from radiology can probably reduce the risk of cancer	☐	☐	☐	☐	☐
d	High quality imaging procedures presuppose clearly specified clinical questions	☐	☐	☐	☐	☐
e	Radiology should more often be included in ordinary health check-ups	☐	☐	☐	☐	☐
f	Radiologists gain professional respect when they actively discuss with and guide clinicians in appropriate use of radiology	☐	☐	☐	☐	☐
g	Patients' confidence in their physician is swayed if the radiologist questions the referral	☐	☐	☐	☐	☐
h	In the referral, physicians should just explain the clinical problem clearly, and not request specific examinations	☐	☐	☐	☐	☐
i	Radiologists must accept that referring physicians and patients may request examinations for other than strictly medical reasons	☐	☐	☐	☐	☐
j	Radiologists have part of the responsibility for limiting the total Norwegian health costs	☐	☐	☐	☐	☐
k	Liberal use of radiology is generally cost-effective	☐	☐	☐	☐	☐
l	Prioritisation based on disease severity, outcome and cost-effectiveness is most relevant in therapy, less in diagnostics	☐	☐	☐	☐	☐

10 Unnecessary use of imaging procedures may have negative consequences. To what extent do you perceive the following consequences to be ethically challenging?

		To a very small extent	To a small extent	To some extent	To a large extent	To a very large extent
a	Reduced access to radiological services for other patients	☐	☐	☐	☐	☐
b	Fewer resources for other health services	☐	☐	☐	☐	☐
c	Unnecessary radiation exposure to patients	☐	☐	☐	☐	☐
d	Unnecessary risk of false positive findings which require medical follow-up	☐	☐	☐	☐	☐
e	Harm the radiologists' professional self-esteem (integrity)	☐	☐	☐	☐	☐
f	Other, specify: ______________________	☐	☐	☐	☐	☐

D Background information

11 Gender Female ☐ Male ☐

12 Are you approved as specialist in radiology? Yes ☐ No ☐

13 Subspecialty/main field of work (please mark only one of the alternatives below):

Angio/inter-vetion	Pediatric radiology	Neuro-radiology	CT	MRI	Ultra-sound	Mammo-graphy	General radiology	Other or additional subspecialty/main field of work:
☐	☐	☐	☐	☐	☐	☐	☐	______________

14 For how many years have you practice as radiologist? ☐

The remaining questions concern only those who practice as radiologist at the moment

15 What percentage of a full-time radiologist position do you work? ____ %

16 How much of your working time (%) is devoted to other work than clinical radiology (research, teaching and administration etc.)? ____ %

17 At what type of institution do you work?

Large public hospital (university or regional) ☐

Small public hospital (community or county) ☐

Private radiological institute ☐

Other, specify: ______________________ ☐

18 How is the capacity of radiology supply within your field of work at your workplace?

Free capacity for receiving more patients ☐

Sufficient according to patient population ☐

Insufficient with long waiting time for patients ☐

19 What is the travel time from your workplace to the closest other radiology service? Approx. _____ hour(s)

Thank you very much for your answers!
