# Supplementary material for: What causes increasing and unnecessary use of radiological investigations? a survey of radiologists' perceptions
Source: BMC Health Serv Res. 2009 Sep 1;9:155. doi: 10.1186/1472-6963-9-155 (PMC2749824; doi:10.1186/1472-6963-9-155)
Supplement: Additional file 3 — Respondents' demographic characteristics and practice information, and some corresponding number in the population. The additional file is a table that displays the respondents' replies on demographic and practice setting variables. In addition, the available corresponding numbers in the population (564 radiologists) is provided. [file 1472-6963-9-155-S3.pdf]

Additional file 3

Respondents' demographic characteristics and practice information, and some corresponding number in the population[1]

Variable	Value	Respondents (n=375) no. (%)	Population (n=564) no. (%)
Gender	Female	161 (42.9)	231 (41.0)
	Male	214 (57.1)	333 (59.0)
Approved specialist / registrar	Approved specialist	312 (83.2)	469 (83.2)
	Registrar	63 (16.8)	95 (16.8)
Years in radiology practice	< 1–9	131 (34.9)	
	10–19	108 (28.8)	
	20–29	65 (17.3)	
	30–40	69 (18.4)	
	Missing	2 (0.5)	
Current employment	Full-time	326 (86.9)	
	Part-time	27 (7.2)	
	Missing	22 (5.9)	
Type of institution employed	Public hospital	311 (88.9)	515 (91.3)
	Large (university or regional) (n = 246)		
	Small (community or county) (n = 60)		
	Unspecified (n = 5)		
	Private radiological institute	39 (10.4)	49 (8.7)
	Other	5 (1.3)	
	Missing	20 (5.3)	
Capacity of radiology supply in own practice	Free capacity for receiving more patients	18 (4.8)	
	Sufficient according to patient population	150 (40.0)	
	Insufficient with long waiting time for patients	172 (45.9)	
	Missing	35 (9.3)	
Travel time from own workplace to closest other provider of radiology services	< 0.5 hour	209 (55.7)	
	0.5 – 1 hour	108 (28.8)	
	> 1 hour	34 (9.1)	
	Missing	24 (6.4)	

[1]Only some of the numbers are available and these are included.
